# Human ability in identification of location and pulse number for electrocutaneous stimulation applied on the forearm

**DOI:** 10.1186/1743-0003-11-97

**Published:** 2014-06-07

**Authors:** Bo Geng, Winnie Jensen

**Affiliations:** 1 Department of Health Science and Technology, Aalborg University, Fredrik Bajers vej 7D, Aalborg, Denmark

## Abstract

**Background:**

The need of a sensory feedback system that would improve users’ acceptance in prostheses is generally recognized. Feedback of hand opening and position are among the most important concerns of prosthetic users. To address the two concerns, this study investigated the human capability to identify pulse number and location when electrical stimulation applied on the forearm skin. The pulse number may potentially be used to encode the opening of prosthetic hands and stimulation location to encode finger position.

**Methods:**

Ten able-bodied subjects participated in the study. Three electrodes were placed transversely across the ventral forearm spatially encoding three fingers (i.e., thumb, index, and middle finger). Five different pulse numbers (1, 4, 8, 12, and 20) encoded five levels of hand opening. The study consisted of three experiments. In the three experiments, each after a training session, the subjects were required to identify among: (a) five stimulation locations, (b) five pulse numbers, or (c) ten paired combinations of location and pulse number, respectively. The subjects’ performance in the three identification tasks was evaluated.

**Results:**

The main results included: 1) the overall identification rate for stimulation location was 92.2 ± 6.2%, while the success rate in two-site stimulation was lower than one-site stimulation; 2) the overall identification rate for pulse number was 90.8 ± 6.0%, and the subjects showed different performance in identification of the five pulse numbers; 3) the overall identification rate decreased to 80.2 ± 11.7% when the subjects were identifying paired parameters.

**Conclusions:**

The results indicated that the spatial (location) and temporal (pulse number) identification performance are promising in electrocutaneous stimulation on the forearm. The performance degraded when both parameters had to be identified likely due to increased cognitive load resulting from multiple tasks. Utilizing the proposed coding strategy in practical prosthetic hands remains to be investigated for clinical evaluation of its feasibility.

## Background

Despite a great deal of progress achieved in control of modern upper limb myoelectric prostheses, development of a system for sensory feedback in the prostheses is still a challenge to be solved. To regain users’ ability to ‘feel’ the environment from their artificial limbs, many research efforts have been dedicated to exploit appropriate techniques to substitute the sensory feedback that is lost in amputees.

Electrocutaneous stimulation appears to be one of the feasible techniques, in which electrical current is passed through the skin to activate the tactile sense [1,2]. Varying an appropriate stimulus parameter may modify the tactile sense. The feedback information can therefore be encoded and conveyed to the user by modulating a stimulus parameter [3,4]. Feedback of force exerted by the artificial limbs has been investigated most often. Force levels were encoded either by modulating pulse rate or amplitude in a linear or nonlinear way [4-6]. Force feedback has been implemented in myoelectrically controlled prostheses for clinical evaluation and the results generally showed positive effects on prosthetic control [7-9].

In comparison to force feedback, feedback of position is relatively less described in arm prosthesis prototypes. Prostheses able to provide position feedback may reduce the amount of attention required to control the device and thus allow for more intuitive grasping. An epidemiologic study emphasized that position feedback is one of the most important aspects to be incorporated in future myoelectric hand prostheses [10]. A few recent studies investigated various strategies for position coding [11-13]. For example, D’Alonzo et al. compared different configurations of electrotactile and vibrotactile stimulation to encode finger position [11]. Witteveen et al. evaluated the feedback on hand opening by activation of one of the electrotactile or vibrotactile stimulators placed on the forearm [12]. Saunders and Vijayakumar used a vibrotactile feedback array and the grip force was translated into a stimulation location with the intention to examine the role of feed-forward and feedback for closed-loop prosthesis control [13].

In this study, the number of pulses in a pulse burst is proposed to encode the hand opening. Previous research found that the number of pulses can effectively modulate the perceived sensation magnitude [14]. The inverse relation between pulse number and perceived magnitude has a low slope (about 1.8), which implies a wider dynamic range of sensations without evoking pain [15]. In addition, modulation of the pulse number is equivalent to modulation of the pulse burst duration given a constant pulse rate. It might make sense for the users to mentally associate the temporal length to the physical length (i.e., the level of hand opening).

Stimulation location is proposed to encode finger position. Spatial geometry of multiple electrodes can be used to encode the location of feedback information. A comparative study revealed that spatial modulation using multiple electrodes was effective and superior to other single electrode codes examined [16]. While some studies investigated the human capability to localize stimulation on the abdomen and fingertips [16,17], the spatial discriminability in the forearm has rarely been studied. Moreover, to our knowledge, there have not been published studies on the localization of two-electrode stimulation on the forearm.

The present study aimed to investigate the human capability to identify stimulation location and pulse number when electrical stimulation is applied to the ventral forearm skin. We examined: (1) human ability to identify among five stimulation locations, each mentally related to one or two fingers, (2) human ability to identify among five different pulse numbers, each mentally related to a level of hand opening (3) human ability to identify among ten paired combinations of stimulation location and pulse number.

## Methods

### Subjects

10 able-bodied subjects (7 males and 3 females, age 25-39 years, mean 29.1 years) participated in the study. All subjects signed an informed consent prior to the experiments. The experimental protocol was in accordance with the Declaration of Helsinki and approved by the North Denmark Region Committee on Health Research Ethics (Approval no. N-20110063). The subjects had no visible skin diseases in the forearm and no known history of neurological disorders.

### Electrode placement

**Figure 1:**
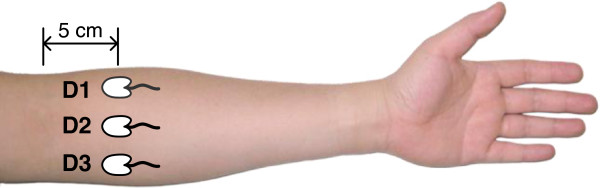
**Electrode placement.** Three self-adhesive solid gel surface electrodes (D1, D2, D3) were transversely placed 5 cm distant from the elbow crease on the ventral side of the left forearm. The three electrode sites were intended to encode the position of three fingers.

Three self-adhesive solid gel surface electrodes (Ambu Neuroline 700, skin contact size 20 mm × 15 mm, ‘duck foot’ shape, silver/silver chloride) were placed 5 cm distally to the elbow crease on the ventral aspect of the left forearm (Figure 1). A return electrode (PALS Platinum: 40 mm × 64 mm, oval shape) was positioned over the dorsal side of the wrist on the same forearm. The center-to-center distance between the electrodes ranged from 40 mm to 50 mm depending on the size of individual forearms. The skin was prepared by gently shaving when needed and moisturizing with a water-soaked cotton cloth to facilitate electrical conductivity.

### Stimulation parameters

A symmetric, biphasic (a negative phase followed by a positive phase), rectangular waveform with a pulse duration of 100 μs for each phase was used. Biphasic pulses were used because they produce a less amount of skin reddening and a more comfortable sensation than monophasic pulses [2]. The shape of the waveform was generated by STG2008 (Multi Channel Systems, Reutlingen, Germany) and a DS5 (Digitimer, Hertfordshire, UK) then translated the voltage output of the STG2008 into an isolated, constant current stimulus. An 11-point numerical rating scale (0 represents no sensation and 10 represents upper limit of the sensation) was used to determine the current level for each subject. The following procedure was performed to ensure clear perception without pain at all the three sites. First, sensation threshold was measured for the three sites since threshold varies with location (for example, 2.8 mA at D1, 2.4 mA at D2, 1.9 mA at D3 in one subject). Then, the current level was tuned above the highest threshold until the subject rated the perceived stimulation with moderate scores (usually 2-5) at the three sites. The current levels for different subjects were between 3 to 4.4 mA depending on individual sensation thresholds.

### Experimental procedure

Three experiments were performed to assess the sensory identification ability of each subject. Throughout the three experiments, the current intensity was constant at which the subject was able to clearly perceive the stimulation at all three locations.

In each of the three experiments, a training session was carried out before assessment of the identification ability. The training aimed to familiarize the subjects with the stimuli and the subjects thus learned to mentally associate different sensations evoked by those stimuli with corresponding stimulation locations or pulse numbers. The training session consisted of two phases: first non-random and then random presentations of stimuli. After the presentation of each stimulus, the subjects orally indicated which stimulus was perceived and the experimenter provided verbal feedback of the correct answer. In both phases, the subjects were trained with at least 50 trials. To maintain alertness and minimize possible sensory adaptation, the subjects were given 10 minutes rest between experiments.

### Experiment 1: Identification of stimulation locations

**Figure 2:**
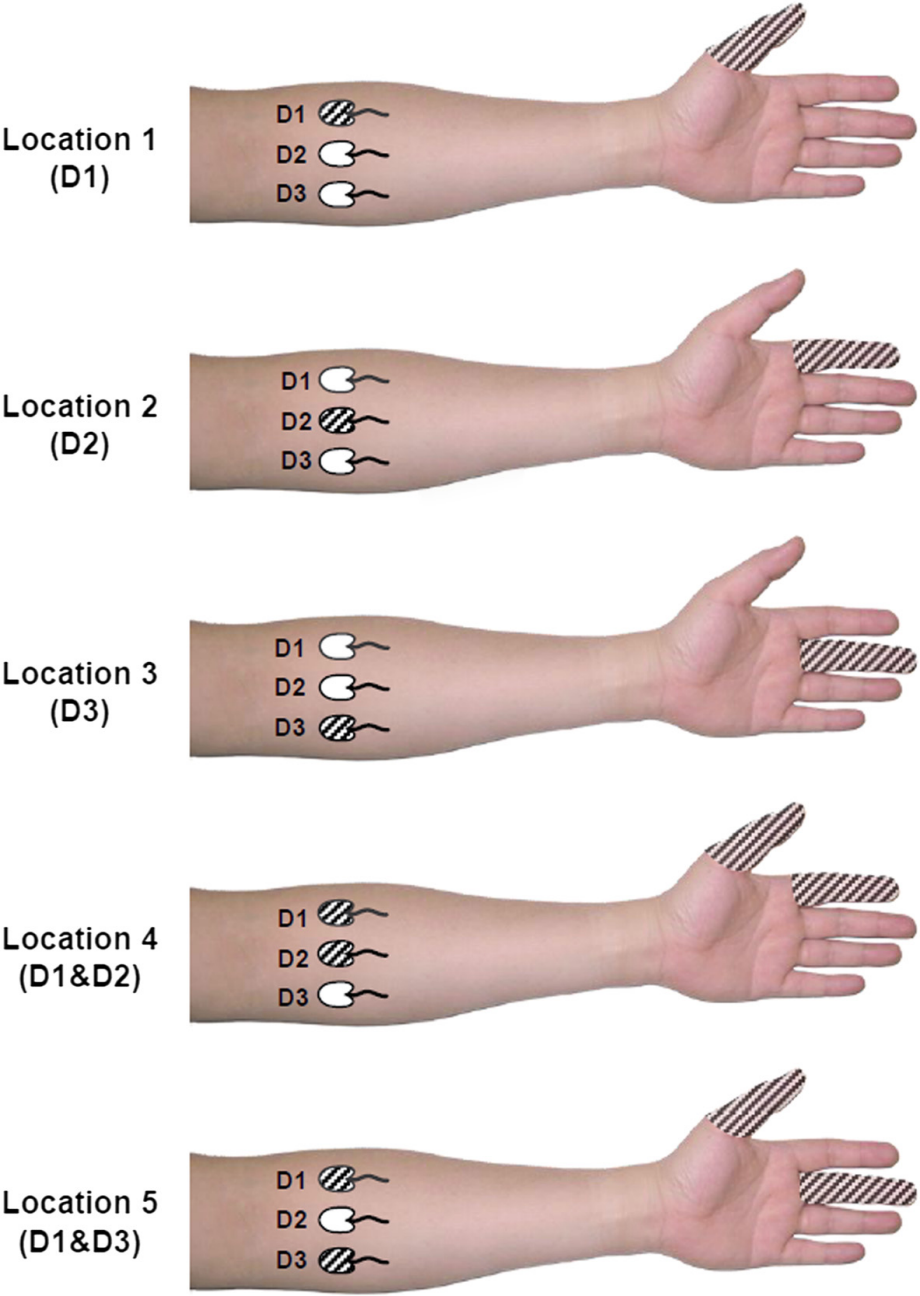
**Stimulation locations and fingers to be mentally associated.** The subjects were instructed to mentally associate the stimulation applied at five locations **(D1, D2, D3, D1 & D2, D1 & D3)** to one or two particular fingers. The shadowed electrodes and fingers represent the association.

In this experiment, stimulation was applied to either a single or a pair of electrode sites (D1, D2, D3, D1&D2, and D1&D3). D1, D2 and D3 represent the thumb, the index finger and the middle finger, respectively. D1&D2 (D1&D3) represents that stimulation was simultaneously delivered through D1 and D2 (D3). The subjects were instructed to mentally associate the perceived stimulation location with the three digits. Figure 2 graphically illustrates the association between the stimulation locations and the fingers. To evaluate the identification ability, for each subject, 50 trials were applied in a random order with each location repeated for 10 times. Once a stimulus was presented, the subject was asked to discern and orally report the linked fingers. All stimuli contained four pulses in this experiment.

### Experiment 2: Identification of pulse numbers

**Figure 3:**
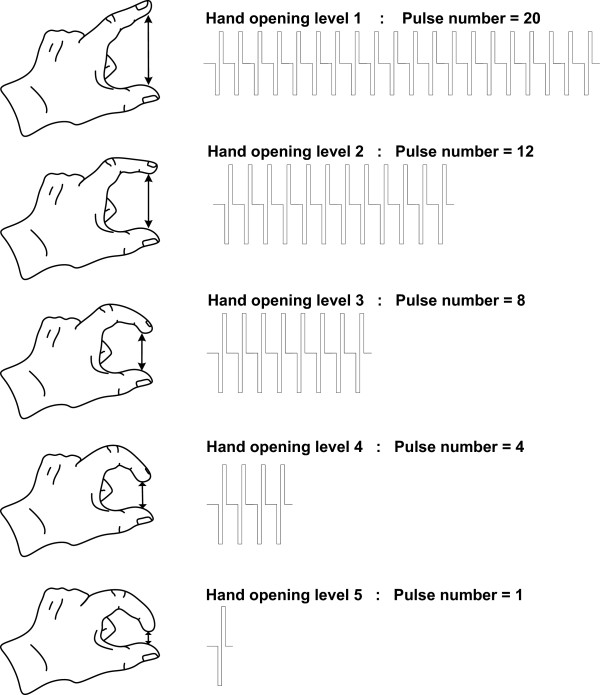
**Pulse numbers and hand opening levels to be mentally associated.** The subjects were instructed to mentally associate five pulse numbers (1, 4, 8, 12, 20) to five levels of hand opening.

In this experiment, the subjects were instructed to mentally associate five different pulse numbers (1, 4, 8, 12, or 20) with five levels of hand opening (or sizes of gripped objects). Figure 3 illustrates the association between the pulse numbers and the hand opening levels. To evaluate the identification ability, for each subject, 50 trials were applied in a random order with each pulse number repeated for 10 times. Once a stimulus was presented, the subject was asked to report the linked level of hand opening. All stimuli were applied at D1&D2 in this experiment.

### Selection of pulse rate and pulse numbers

**Figure 4:**
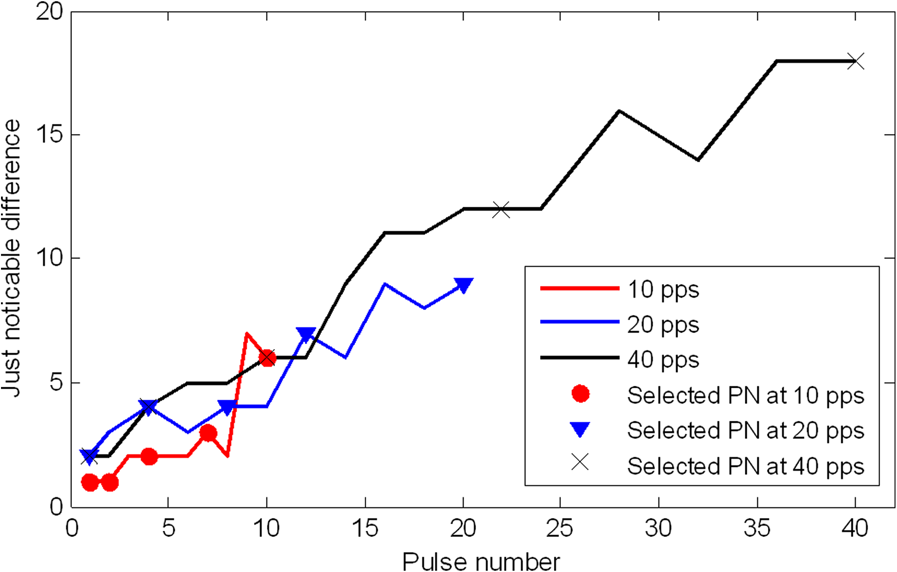
**Just noticeable difference (JND) for pulse number.** The curves of JND for pulse number were drawn based on the measurement at three pulse rates: 10, 20, 40 pps in one subject. A group of five pulse numbers was selected for each pulse rate, ensuring that the ‘spacing’ between two successive pulse numbers equal to or larger than the JND. The three groups of selected pulse numbers were marked on the three curves, respectively.

Since the choice of pulse rate and the ‘spacing’ between two successive pulse numbers could highly influence the performance in pulse number identification, the ‘optimal’ pulse rate and pulse numbers were selected in preliminary experiments. The pulse rates of 10, 20, and 40 pulses per second (pps) were tested and compared. Low pulse rates were considered because high pulse rates have previously been reported to be less clear and harder for the subjects to interpret [18].The selection of ‘optimal’ pulse numbers was based on a method using just noticeable difference (JND) of pulse numbers. The JND of a specific pulse number was determined using the following method: (1) A pair of stimuli was presented in sequence with the first as the baseline stimulus and the second having a greater pulse number; (2) After each stimulus pair presented, the participant was asked to report whether he perceived the difference between the two stimuli or not; (3) The second stimulus increased until the participant detected the difference; (4) The difference in pulse number between these two stimuli was then recorded as the JND of the base stimulus. JNDs of a range of pulse numbers were measured for each of the three pulse rates. That is, JNDs of pulse number 1, 2, 3,…, 10 for pulse rate 10 pps (i.e., totally 10 JNDs obtained), JNDs of pulse number 1, 2, 4, 6, …, 20 for pulse rate 20 pps (i.e., totally 11 JNDs obtained), JNDs of pulse number 1, 2, 4, 6, …, 20, 24, 28,…, 40 for pulse rate 40 pps (i.e., totally 16 JNDs obtained). Five pulse numbers were selected for each pulse rate, according to: (a) PN1 was always equal to 1, (b) PN5 equal to the maximum pulses in one second (i.e. the pulse rate), and (c) PN2 ≥ PN1 + JND (PN1), PN3 ≥ PN2 + JND (PN2) and so on. The selection criterion was that the five values distributed within one second and meanwhile their spacing equal to or larger than the JNDs. This JND-based method ensured the selected pulse numbers were theoretically distinguishable. Figure 4 shows the measured JNDs and selected pulse numbers for each of the three pulse rates.

As such, three groups, each consisting of five pulse numbers, were selected and then evaluated in the subject. The pulse rate and the corresponding group of pulse numbers resulting in the best identification rate was chosen for evaluation in more subjects. Table 1 shows the three groups of selected pulse numbers and respective identification correct rate for each pulse rate. It is noteworthy that this procedure for selection of ‘optimal’ pulse numbers was based on the evaluation of only one subject. There were likely subject variances in JNDs, which might have an impact on the choice of pulse numbers. Besides, in the procedure of measuring JNDs, for practical reason the order of the baseline and test stimuli was always the same and known to the subjects, which might be prone to subject bias.

**Table 1:** Selected pulse numbers and identification accuracy at three pulse rates

**Pulse rate**	**Pulse numbers**	**Accuracy**
**10**	1, 2, 4, 7, 10	77.5%
20*	1, 4, 8, 12, 20	87.5%
**40**	1, 4, 10, 22, 40	82.5%

*: This pulse rate and the group of pulse numbers were chosen.

### Experiment 3: Identification of combination of location and pulse number

In this experiment, the subjects were instructed to identify not only the stimulation location but also the pulse number. To avoid too many combinations, only D1&D2 and D1&D3 were selected to combine with five pulse numbers (1, 4, 8, 12, or 20). Thus, 10 paired combinations of pulse number and location were generated. To evaluate the identification ability, for each subject, 50 trials were applied in a random order with each combination repeated for 5 times. The subject’s response was considered correct only if both parameters were correctly recognized.

### Statistical analysis

The performance metric was the identification rate, defined as the percentage of stimuli successfully recognized by the subjects. A paired two-tailed t-test was used to compare between the performance in identification of one-electrode stimulation and two-electrode stimulation (Experiment 1). A one-way repeated measures ANOVA was used to compare the success rate in identification of five individual pulse numbers (Experiment 2). Multiple comparisons were subsequently performed using Holm-sidak test to identify pairwise significance. A paired t-test was also used to compare between the success rates of identifying stimulation location alone (Experiment 1) or pulse number alone (Experiment 2), and their marginal success rate in the combination case (Experiment 3).

## Results

### Ability to identify stimulation locations

**Figure 5:**
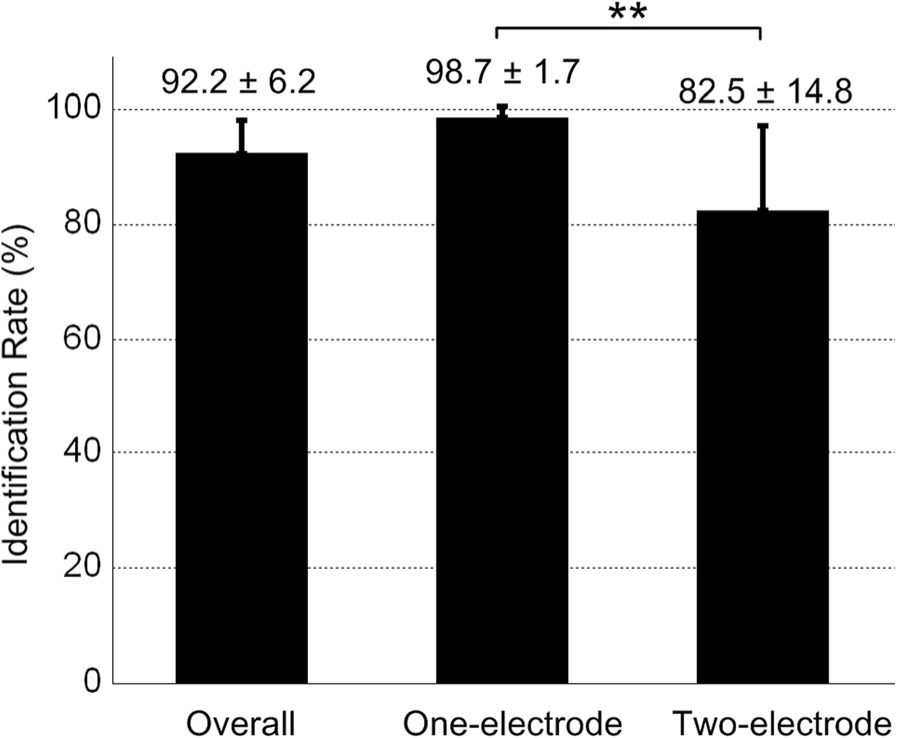
**Identification rate (mean ± standard deviation, n = 10) for stimulation location.** The three error bars show the overall identification rate, the identification rate in one-electrode stimulation, and in two-electrode stimulation, respectively. ** indicates a significant difference (*p* < 0.01).

Figure 5 shows the identification rate for stimulation location. The overall success rate in identification among the 5 stimulation locations (D1, D2, D3, D1&D2, D1&D3) was 92.2 ± 6.2%. The identification rate in two-electrode stimulation (82.5 ± 14.8%) was lower than that in one-electrode stimulation (98.7 ± 1.7%). A paired t-test indicated a statistically significant difference (*p* < 0.01) between the two stimulation scenarios. Table 2 shows in details the percentages of reported location and the location where the stimuli were actually delivered.

**Table 2:** Percentage of reported and actually stimulated location

**Stimulated location**			**Reported location**		
	**D1**	**D2**	**D3**	**D1&D2**	**D1&D3**
**D1**	99%	1%	0	0	0
**D2**	2%	97%	0	1%	0
**D3**	0	0	100%	0	0
**D1&D2**	7%	2%	0	78%	13%
**D1&D3**	2%	0	2%	9%	87%

### Ability to identify pulse numbers

**Figure 6:**
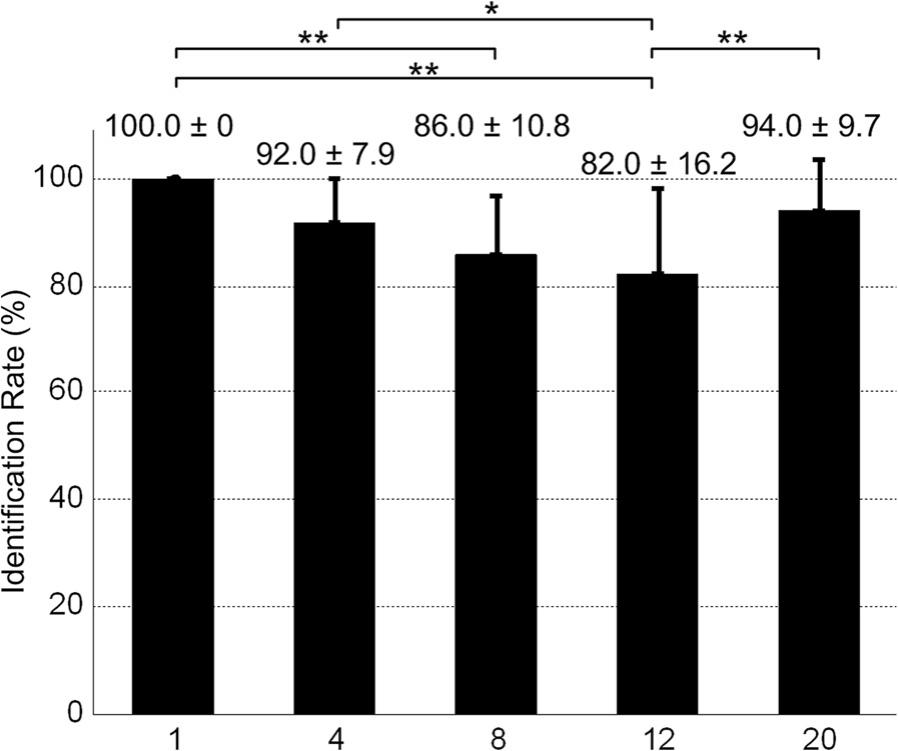
**Identification rate (mean ± standard deviation, n = 10) for pulse number.** The error bars show the identification rates for five individual pulse numbers. ** indicates a significant difference (*p* < 0.01). * indicates a significant difference (*p* < 0.05).

The overall identification rate for the five pulse numbers (1, 4, 8, 12, 20) was 90.8 ± 6.0%. However, the subjects’ capability differed in identification of the five pulse numbers (as shown in Figure 6). Identification rate for one pulse was 100% with all 10 subjects, while identification of 12 pulses appeared most challenging (82.0 ± 16.2%). The results of repeated measures ANOVA test showed a significant difference in the performance of identifying the five pulse numbers (*p* < 0.01). Multiple comparisons indicated significant difference between the following pulse number pairs: 1 and 8 (*p* < 0.01), 1 and 12 (*p* < 0.01), 4 and 12 (*p* < 0.05), 12 and 20 (*p* < 0.01). Table 3 lists in details the percentages of reported or decoded hand opening level and the pulse number actually delivered.

**Table 3:** Percentage of decoded hand opening level and pulse number delivered

**Pulse number**			**Opening level**		
	**Level 1**	**Level 2**	**Level 3**	**Level 4**	**Level 5**
**1**	100%	0	0	0	0
**4**	1%	92%	7%	0	0
**8**	0	6%	86%	8%	0
**12**	0	0	16%	82%	2%
**20**	0	0	0	6%	94%

### Ability to identify combined parameters

**Figure 7:**
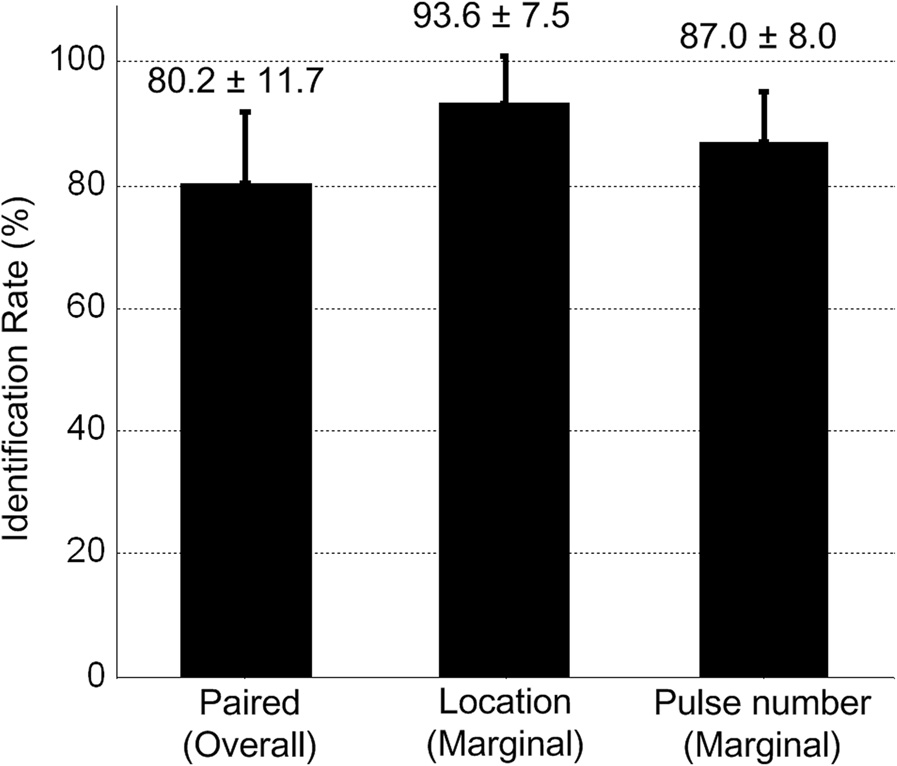
**Identification rate (mean ± standard deviation, n = 10) for paired parameters.** The error bars show the identification rate for paired parameters, as well as their marginal success rates.

Figure 7 shows the success rate in the case of identifying combined parameters. The overall accuracy in identification of paired combinations was 80.2 ± 11.7%. The marginal identification rates (i.e., success rate for stimulation location or pulse number regardless of the other) are 93.6 ± 7.5% for location and 87.0 ± 8.0% for pulse number, respectively.

To examine if the performance became worse when two parameters needed to be identified, the identification rate for location alone (82.5%) in Experiment 1and pulse number alone (90.8%) in Experiment 2 were compared with their marginal success rate in combination case. The results showed that combination lowered the success rate in pulse number identification by 3.8% with statistical significance (*p* = 0.02). On the contrary, the accuracy in location identification was improved by 11.1% without showing significant difference (*p* = 0.06).

### Between- and within-subject variability

**Figure 8:**
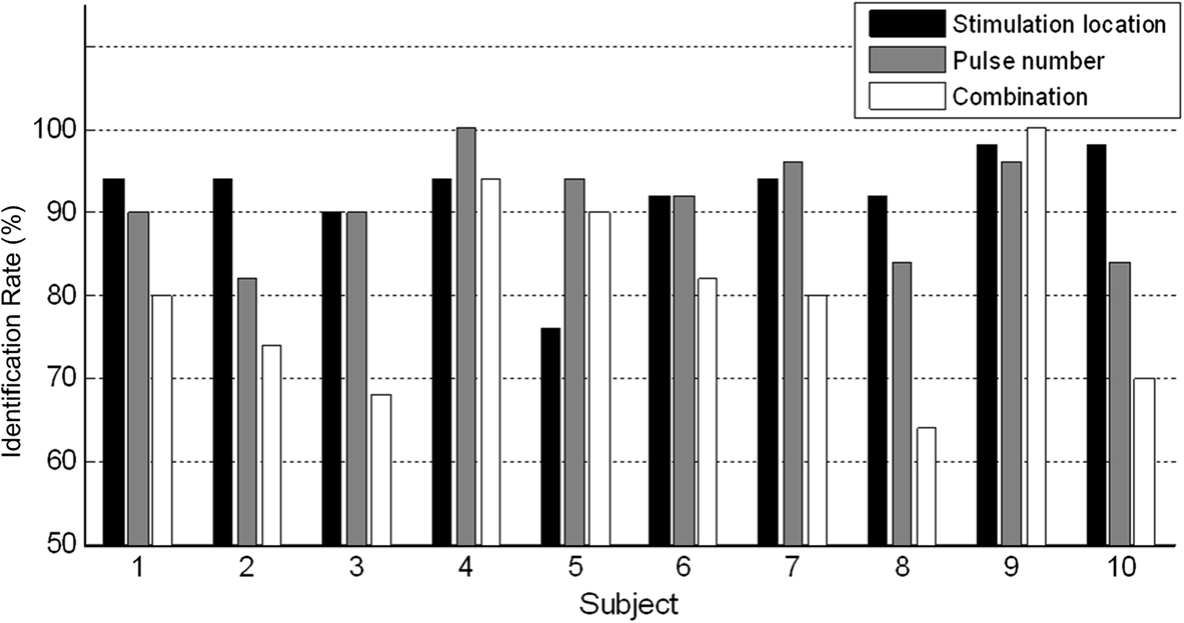
**Identification rate for 10 individual subjects.** The bar plot shows the identification rate obtained from the three experiments for 10 individual subjects.

Figure 8 shows the identification rates in the three experiments for 10 individual subjects. The between subject variability was measured by calculating the standard deviation of the correct identification rate measured in the three experiments, which are 6%, 6%, and 12% respectively. Identification of paired parameters showed a considerably higher variability than identification of a single parameter, reflecting the subjects’ perceptual difficulty and fluctuation in performing more complex identification tasks.

The within subject variance appeared consistent in most cases, which indicated good reliability. That is, better and comparable identification rates were observed in location and pulse number identification tasks, and a lower accuracy in identification of paired parameters. There were only a few exceptions. For instance, subject 5 was good in identification of pulse number and combined parameters, but not in identification of location, and subject 9 performed excellently in all three experiments. This might be due to subject-to-subject physiological variance or difference in learning rate.

## Discussion

### Spatial (location) identification

In the experiments reported in this paper we have assessed the human ability to discriminate stimulation locations. Three electrodes were placed on the ventral forearm because the ventral side bears significantly lower perception threshold than the dorsal side according to our previous findings [19]. This implies better power efficiency, which is important for the application in prosthetic devices. Moreover, electrocutaneous stimulation of the ventral forearm more easily elicits comfortable touch sensation, rather than tingling, pricking, or other paraesthesia than the dorsal side [14].

The ability to localize stimulation highly depends on where the stimulation is applied on the body surface as well as the inter-electrode distance [20]. In our experiments, the inter-electrode distance was chosen to be 40-50 mm, which ensured that the spacing of the electrodes is greater than the two-point discrimination threshold (i.e., the minimum distance at which two points of stimulation are detected, about 9 mm in the forearm). The two-point discrimination threshold is also a function of frequency, pulse width and stimulation technique [21,22].

A larger number of sites may increase the complexity of the identification tasks and have an impact on the users’ performance. In D’Alonzo’s study, five sites on the forearm of health subjects were used to encode five fingers [11]. The results showed a lower identification rate (i.e., 94% *vs*. 98.7% in single-site identification and 79% *vs*. 82.5% in multi-site identification), perhaps partly due to increased complexity of identification tasks.

### Crosstalk in two-electrode stimulation

Since the inter-electrode distance in our experiments was greater than the two-point discrimination threshold in the forearm for electrocutaneous stimulation (approximately 9 mm [21]), the subjects theoretically could localize the stimulation with high success rate in both one-electrode and two-electrode stimulation. However, the identification rate in two-electrode stimulation was not as good as in one-electrode stimulation likely because the crosstalk between channels interfered the subjects’ perceptual experience. As shown in Table 2, the most frequent misidentifications are between D1&D2 and D1&D3, which to some extent reflects the influence of crosstalk on the subjects’ discriminability.

In addition, the same current amplitude was applied at the three locations for each subject. To ensure clear perception of the stimulation at all three locations, the current amplitude was chosen to be sufficiently high (3-4.4 mA depending on individual perceptions). Since the sensation thresholds at the three sites are different [19], the perceived magnitude for the same current amplitude might have been different at the three sites. That is, the subjects perceived higher intensity at the site characterized with lower perception threshold and vice versa. It means that at the site with lower sensation threshold a lower current level is sufficient to elicit a clear perception. Therefore, applying appropriate current level taking the threshold into consideration may be able to reduce the required current amplitude and consequently reduce the crosstalk between channels.

### Temporal (pulse number) identification

As the pulse rate was constant, stimulation with more pulses produced longer perception duration. Hence, Experiment 2 partially assessed the subjects’ capability to distinguish among different time durations of perception (i.e., temporal identification). Successive pulse numbers, i.e., 8 and 12 pulses as well as 4 and 8 pulses, were most frequently misidentified likely because their temporal difference (i.e., difference in the pulse train duration) was not sufficiently large so that it did not exceed the JND for all subjects.

The sensation magnitude may also play a role in discrimination of pulse numbers. Previous study shows that the pulse number effectively modulated the perceived magnitude [14]. Greater number of pulses could elicit stronger sensation intensity. Sensation magnitude may thus be one of the perceptual dimensions used by the subjects to identify between the five pulse numbers.

In addition, some subjects reported that they seemed able to count the pulses when the pulse number was small, whereas it became difficult in the case of a greater number of pulses. It implies that the subjects distinguished pulse numbers not only based on the pulse train duration and sensation magnitude, but also partially based on the number of pulses perceived. This may account for the 100% accuracy in identification of one pulse.

### Paired parameter identification

In Experiment 3, identification of paired parameters degraded the performance as expected. Similar outcome was reported in a study on vibrotactile stimulation on the forearm, a combination of site and force discrimination led to a significantly lower recognition rate (78%) than discrimination of a single parameter (93%) [23]. Composite nature of multiple tasks increased the cognitive loads and consequently resulted in a higher discrimination difficulty. When the subjects need to make multiple decisions (i.e., select the location and select the pulse number), each of the subtasks is characterized with a certain success rate, where the success rate for identification of one parameter might depend on the other. A further investigation of this interaction needs to determine the baseline success rates for each subtask.

Although the overall performance was worse in paired parameter identification, the marginal success rate in spatial identification was actually improved by 11.1% in Experiment 3. The improvement might be either due to only two locations involved (D1&D2 and D1&D3), or because learning of location was relatively more robust to the interruption from a second subtask. In other words, spatial modulation might be easier for the human subjects to learn than temporal modulation.

### Practical relevance in prostheses

In multi-fingered prostheses, it is natural to use five sites to encode five fingers. In our study, three instead of five sites were chosen out of the consideration that the first three fingers are most frequently used, and majority of daily grasping and lifting tasks can be accomplished by the three fingers. Moreover, the three electrodes were positioned transversely on the ventral side of the forearm with the intention to resemble the biological spatial organization of the three associated fingers. This arrangement might provide more natural sensory feedback and enhance the body awareness of the prostheses, although wider electrode spacing may improve the identification rate.

The pulse number was proposed to encode the object size (or hand opening) with their perceptual relevance taken considered, i.e., physical length substituted by temporal length. The relevance might reduce the subjects’ conscious burden of interpreting the code. However, there is an issue of time delay when feedback information is encoded by a time-dependent parameter such as pulse number. Therefore, the determination of the time spanning of the code is critical to avoid unacceptable delay of the feedback. In our study, 5 levels of hand opening were encoded by 5 pulse numbers with the level 5 corresponding to the greatest pulse number lasting 1 second. Although existing literature estimated that the optimal controller delays (i.e., the amount of time between the user’s command and the actuation of the device) may range from 50 to 400 ms to maintain acceptable prosthesis control and prevent a noticeable delay [24], whether and how 1 s time duration for feedback of hand opening would affect the prosthetic usability remains to be assessed by psycho-physiological examinations.

In addition to the application in improving daily control of prosthesis, the proposed feedback can also be intended to promote the embodiment of prostheses, or treat phantom limb pain through the use of prosthetic devices. In these cases, the time delay of the feedback may not be as critical.

### Translation to amputee subjects

It should be noted that the sample population is not representative of upper-limb amputee patients. Therefore, the results ought to be interpreted with caution before application in amputee patients because when stimulating the damaged limb, the perceptual experience may considerably differ from those in able-bodied subjects. In the cases where mapping of the phantom hand on the residual limb occurs, amputee patients could be expected to achieve better identification performance. On the other hand, majority of amputees are interfered by phantom pain or stump pain, which may negatively affect their ability in sensory identification. Whether similar results can be obtained with amputees, and in what degree the identification ability is affected by pain need to be investigated in future work.

## Conclusions

The capability of a human to identify location and pulse number of electrocutaneous stimulation was evaluated in able-bodies subjects. Both achieved promising identification performance (92.2% and 90.8%). The spatially distributed afferent information may be used in sensory feedback systems endowed in multifingered prostheses. The pulse number may be varied to encode the level of hand opening by substitute of physical length to temporal length. To validate the efficacy of the proposed coding in control of a prosthesis device, future work is to be planned.

## Competing interests

The authors declare that they have no competing interests.

## Authors’ contributions

BG and WJ outlined the fundamental concepts of the scientific research. BG designed the experimental protocol and performed the experiments. BG conducted data collection and analysis. BG and WJ contributed to the preparation of the manuscript. Both authors read and approved the final manuscript.
